# Development and Validation of the Social Network Addiction Scale (SNAddS-6S)

**DOI:** 10.3390/ejihpe10030056

**Published:** 2020-07-26

**Authors:** Esther Cuadrado, Rocío Rojas, Carmen Tabernero

**Affiliations:** 1 Maimonides Biomedical Research Institute of Cordoba (IMIBIC), 14004 Córdoba, Spain; carmen.tabernero@usal.es; 2 Department of Psychology, University of Córdoba, 14071 Córdoba, Spain; rociors1995@gmail.com; 3 Department of Psychology, University of Salamanca, 37007 Salamanca, Spain

**Keywords:** social network addiction, scale development, scale validation, confirmatory and exploratory factor analyses

## Abstract

The use of social networks has increased exponentially, especially among youth. These tools offer many advantages but also carry some risks such as addiction. This points to the need for a valid multifactorial instrument to measure social network addiction, focusing on the core components of addiction that can serve researchers and practitioners. This study set out to validate a reliable multidimensional social network addiction scale based on the six core components of addiction (SNAddS-6S) by using and adapting the Bergen Facebook Addiction Scale. A total of 369 users of social networks completed a questionnaire. Exploratory and confirmatory factor analyses were performed, and different competing models were explored. The external validity of the scale was tested across its relations with different measures. Evidence for the validity and reliability of both the multidimensional SNAddS-6S and the unidimensional Short SNAddS-6S was provided. The SNAddS-6S was composed of 18 items and five different factors (time-management, mood modification, relapse, withdrawal, and conflict), with the time-management factor as a higher-order factor integrated by salience and tolerance as sub-factors. The Short SNAddS-6S was composed of six items and a unifactorial structure. This scale could be of relevance for researchers and practitioners to assess the extent to which individuals suffer from social network addiction and to study the potential predictors and risks of such addiction.

## 1. Introduction

Social networks (SN) are attractive entertainment tools, especially for youths [1,2,3]. New technologies are integrated into their lives, so it would be impossible for individuals to imagine their existence without SN [1]. The attractiveness of SN lies in the many possibilities they offer—easy and real-time communication, sharing photos and videos, identity construction, etc. [4,5,6].

At the same time, SN have some risks—bullying, the loss of intimacy, the possibility of developing social network addiction (SNA), etc. [7]—and its abuse might involve physical, cognitive, and emotional problems [1,8,9,10].

In their conceptualization of SNA, Andreassen and Pallesen [11] noted the six symptoms that necessarily appear in all kinds of addiction (both chemical and behavioral), as described by Griffiths [12]. On the other hand, the Diagnostic and Statistical Manual of Mental Disorders [13] does not include SNA as an addictive disorder; however, a body of studies supports the existence of this addiction [14]. People between 16 and 31 years old spend more and more time surfing the net and (ab)using SN [15]. SN are attractive, act as immediate reinforcers, and present ease of access, all of which are key factors in any addiction [16]. While non-addicted people also use the Internet for social functions, and while social purposes are not a pre-requisite for addiction, it may be observed that addicted people use the Internet more for social functions than non-addicted people [17]. Nevertheless, it is difficult to draw the map of prevalence of SNA due to the types of studies performed—usually non-representative student samples, with different screening methods and cut-off points [18]. Thus, more studies are needed on this topic.

As far as we know, currently only the Bergen Social Media Addiction Scale (BSMAS) measures a very similar concept to SNA, focusing on the six core components of addiction [8,19]. This instrument represents an adaptation of the Bergen Facebook Addiction Scale (BFAS) and contains six items based on the components of addiction outlined by Griffiths [12]. Although an extensive validation of the scale was not performed during the aforementioned studies, some psychometric data for this scale were presented. Multiple language adaptations of the BSMAS were made, being available, among others, in Italian [20] and Persian [21], but as far as we know, there is no Spanish version. Moreover, although the BSMAS and its multiple language adaptations focus on the six core components of addiction, they only contemplate six items, one for each of the six core components of addictions. This means that the BSMAS is presented as a six-item unifactorial scale. Thus, in this form, this scale does not allow professionals and researchers to work independently of each of the six core components of SNA. For this reason, in this study we intend to develop and validate a Spanish multifactorial scale with three items for each of the six core components of SNA. The advantage will be the obtention of a multifactorial scale validated in Spanish that will allow researchers and professionals not only to measure SNA, but also to investigate each of the six core components of SNA. Moreover, in the BSMAS, the authors focused on “social media” and not on SN; nevertheless, social media and SN are not the same [1]. Social media refers to the possibility to produce and share content online, while SN refers to virtual communities that allow users to create profiles and interact online with other people [1]. In this study, we are focusing on SNA, acknowledging SN as a specific type of social media use. In this sense, SN imply interconnectedness between people [1] and an “always on” lifestyle, as well as a way of being and relating, referring to how we are, how we introduce ourselves, and how we relate to other people [1]. Then, SN fulfill basic human needs such as the need to belong [22], identity construction [5], social support, and expression [23]. Accordingly, we are not addicted to the technology [1,24,25] (that is only the medium or tool that individuals use to engage in social networking, gaming, and other particular behaviors), but to interacting and connecting with people and to the positive feelings produced by the “likes” and approvals made by individuals pertaining to our SN [1]. Consequently, SN may be potentially addictive [1,5]. In this sense, and taking into account that interconnectedness is the key function of SN, Kuss and Griffiths [1] have argued that “social networks addiction” may be the appropriate denomination. As such, it was the terminology we have used in our scale.

However, there are other similar scales that are supposed to specifically focus on SNA, but not in the way we want to focus on this study. For example, the Social Networks Addiction Scale (SNAS) measured attitudes toward SN and not addiction as such [4]. Another recently validated SNA scale [26] did not represent the six core components of addiction but only three factors: Control difficulty, negativity in social relations, and decreased functions. While those scales are of interest, the core components (or symptoms) of addiction are not measured. This underscores the need for a reliable and valid instrument for the measurement of SNA based on the symptoms of addiction, and that allows the measurement and analysis of each of the six components separately. Thus, the main aim of this study was to develop and validate a multifactorial SNA scale that reflects those symptoms.

### 1.1. Social Network Addiction: Concept and Measurement

Griffiths’ component model of addiction [12] highlighted that both chemical and behavioral addictions are composed of six core components: Salience, mood modification, tolerance, withdrawal, conflict, and relapse. These six components should be present to determine that the person suffers from addiction. Thus, SNA should present those six symptoms. Subsequently, for individuals with SNA, using SN should be their biggest concern and highest priority motivation (salience), as it changes their mood by exciting or relaxing them (mood modification). Moreover, individuals addicted to SN should want to use SN more and more (tolerance), spending so much time to the point of impairing other social and normal activities (conflict) and suffering psychological and physical symptoms when they cannot use the SN (withdrawal). Lastly, when individuals addicted to SN finally get control of their use, using them again should lead to a relapse of addiction (relapse).

Accordingly, an SNA instrument should also reflect those six components of addiction. Based on the component model of addiction [12], Andreassen et al. [27] developed the BFAS initially with 18 items (three items for each of the six core components of addiction), but retaining a single item for each of the six core components of addiction. The BFAS items were worded according to the criteria of diagnostic addiction, and the BFAS obtained good psychometric properties [27]. To generate our SNA scale (SNAddS-6S), we used the 18 items created by Andreassen et al. [27] and changed the word “Facebook” to “Social Networks.” We expected that the SNAddS-6S would have a factorial structure with six factors representing the six core components of addiction (Hypothesis 1). Moreover, we expected that when retaining the items with higher loading in each factor, the resulting six-item scale (Short SNAddS-6S) would show a unifactorial structure (Hypothesis 2).

### 1.2. Social Network Addiction and Related Variables

Several authors have argued that the abuse of SN can lead to addiction [28]. The expansion of SN can involve a risk to their addictive use [29]. Due to the intrinsic relation between abuse and addiction [30], we expected to find a positive correlation between the SNAddS-6S (and each of the six factors) and the abuse of SN.

Another variable potentially related to SNA is social anxiety. SN can be conceived as a protective barrier to interaction problems and social anxiety, as the mediation of computer communication reduces the fear of the interaction and evaluation [31,32]. Moreover, people with high levels of social anxiety are more likely to be addicted to the Internet, Facebook, and mobile phones than people with low levels of social anxiety, likely because virtual socialization eliminates the physical symptoms produced by social anxiety [33,34,35].

Loneliness and problematic use of the Internet are also related [36,37]. People who feel lonely use the Internet as a way of escaping from everyday life. Internet addiction increases the feeling of loneliness since social network users prefer the use of SN to communicate with other people rather than face-to-face interactions because they find it easier to express themselves online [38]. 

A meta-analysis [39] highlighted that Internet addiction is related to dissatisfaction with life in general, reporting Internet users’ lower levels of both subjective (life satisfaction) and objective (quality of environmental conditions) quality of life. As with other addictive behaviors (gambling, sex, work, etc.), subjective well-being is negatively affected by Internet addiction [40], and smartphone addiction has been related to (dis)satisfaction with life [41]. Moreover, different studies have identified a positive relationship between abuse of the Internet and different psychological indicators like depression, anxiety, sleep disturbance, and social disfunction, all of which are associated with worse subjective well-being [42]. Indeed, loneliness, life satisfaction, and self-esteem were predictors of Internet addiction [37]. Addictive use of social media was also related to life satisfaction [43]. Finally, in a systematic review of Bergen addiction scales, the authors [44] find that almost all the works included in their study (except one) have demonstrated a significant association between the BSMAS and well-being.

Self-esteem is also potentially related to behavioral addictions related to the Internet [37]. It was found that low self-esteem was related to Internet addiction [37], to the use of Facebook [45], and to the use of SN [9,37,43].

In line with these findings, we expected our SNAddS-6S and its different factors to be positively related to social network abuse, loneliness, and social anxiety (Hypothesis 3) and negatively related to life satisfaction and self-esteem (Hypothesis 4).

Finally, regarding socio-demographic factors, women seems to (ab)use the Internet for social interactions more than men [46] and are more prone to behavioral addiction involving social interaction than men [27]. Then, we expected higher scores on the SNAddS-6S and its different factors for women (Hypothesis 5).

## 2. Materials and Methods

### 2.1. Participants

A sample of 369 users of SN (67% female, 33% male), on average 21.82 years old (SD = 3.65, age range = [18, 40]) completed the questionnaire. Most of the sample had a level of university (53.9%) or secondary (38.3) education while 7.8% had primary education. Finally, 56.1% had a partner.

### 2.2. Procedure

Approval from the Cordoba Research Ethics Committee was obtained. After the participants gave their informed consent, they completed an anonymous and confidential online questionnaire hosted on various SN. The questionnaire was shared by the researchers and a master’s thesis student on their SN (WhatsApp, Facebook, Instagram). Both participation and diffusion of the questionnaire were solicited. The questionnaire took approximately 10 min to complete. 

### 2.3. Measures

Participants responded to all of the psychosocial measures on five-point Likert scales.

#### 2.3.1. Social Networks Addiction

To measure the extent to which participants engage in addictive behavior toward SN (using them compulsively, pathologically, and without self-control), we adapted the 18 original items that Andreassen and colleagues used to develop their final six-item BFAS [27] by changing the term “Facebook” to “Social Networks.” This modification of the BFAS was largely the same as the modification that the authors made to construct their BSMAS [19], but in the present study we used the 18 original items used by Andreassen and colleagues to construct their final six-item BFAS scale [27]. In the BSMAS, in contrast, the authors used only the final six items of the BFAS. Moreover, in the BSMAS, the authors used the term “Social Media.” On the basis of the argument that social media and SN are not the same (social media refers to the possibility to produce and share content online, while SN refers to virtual communities that allow users to create profiles and interact online with other people) [1], for our scale we used the more specific term “Social Networks.” The resulting instrument of the present study was called the Social Networks Addiction Scale-6 Symptoms (SNAddS-6S) and was the subject of validation in this study. The Spanish form used in this study can be found in Supplementary Table S1.

#### 2.3.2. Social Network Overuse

To measure participants’ overuse of SN, a behavioral frequency scale was created for the purposes of the study. The four items (“The first thing I do when I get up is look at social networks”; “The last thing I do when I go to bed is look at social networks”, “I usually eat with my mobile nearby to check social networks”, and “When I go out with my friends, we usually carry our mobile phones and check Social Networks while we are together”) created reflected abusive behaviors that can be carried out by SN users. The items were constructed to capture the behavioral pattern of being “always online,” “always-on-and-connected,” and always checking SN [47,48]. Reliability was good (Cronbach’s α = 0.72).

#### 2.3.3. Social Anxiety 

To measure participants’ concerns of feeling judged by others or ashamed in social situations, a brief form (10 items; e.g., “I worry about what others say about me”) of the Spanish version of the Social Anxiety for Adolescents Scale [49] was used. Reliability was high (Cronbach’s α = 0.90).

#### 2.3.4. Loneliness

To measure participants’ feelings of loneliness, a short (five items; e.g., “I lack companionship”) version of the Revised UCLA Loneliness Scale [50] was used. Reliability was good (Cronbach’s α = 0.81).

#### 2.3.5. Life Satisfaction 

To measure global cognitive judgments of one’s life satisfaction, the five-item (“In most ways my life is close to my ideal”) Satisfaction with Life Scale [51] was used. Reliability was high (Cronbach’s α = 0.85).

#### 2.3.6. Self-Esteem

To measure the extent of participants’ self-esteem, the abbreviated (seven items; e.g., “I feel that I am a person of worth, at least on an equal plane with others”) form of the Rosenberg Self-esteem Scale used by Marsh [52] was applied. Reliability was high (Cronbach’s α = 0.93).

### 2.4. Statistical Analyses

First, to test for plausible bias due to common method variance (CMV), Harman’s single-factor test was performed. To this end, an exploratory factor analysis (EFA) was performed by introducing all the items of understudy variables (SNA, social network overuse, social anxiety, loneliness, life satisfaction, and self-esteem) and running a principal component analysis. In the first step, we observed how many factors emerged in the unrotated factor solution to assess the number of factors that could cause the variance in the variables. Because six variables were introduced, with one (SNA) expected to have six factors, we expected to find between six and 11 factors that could cause the variance in the variables. In any case, the presence of CMV would be confirmed if a single factor emerges or if one general factor leads to the majority of the covariance [53]. Then, the same test was performed but by extracting one unique factor. In this case, the presence of CMV will be evident if the percentage of the variance explained by the unique factor extracted is equal to or greater than 50%.

To validate rigorously new measures, EFA should be first performed, and then, with a different sample, the analyses should continue with a confirmatory factor analysis (CFA) [54]. Subsequently, the general sample was randomly divided into two samples. In the first stage, with the first split sample (182 participants), we first explored the suitability for inclusion of the items in further analyses by carrying out preliminary data checks. Items displaying poor (*r* < 0.20) correlations with half of the items of the scale or more were removed. Items that considerably reduced the Cronbach’s alpha values and items with low item-to-total correlations (*r* < 0.25) were removed for further analyses. Moreover, items with large amount of missing data (more than 10% non-responses) were removed. Then, we performed an EFA with varimax rotation to identify factors within the item pools and to exclude items that did not group in conceptually sound factors. Bartlett’s test of sphericity (BTS) and the Kaiser-Meyer-Olkin (KMO) statistic were carried out to assess the suitability of using factor analysis [55].

In the second stage, we carried out a CFA with the second split sample (189 participants) by testing the chi-square (χ^2^), the goodness-of-fit index (GFI), the adjusted goodness-of-fit index (AGFI), the comparative fit index (CFI), the root-mean-square error of approximation (RMSEA), and the Akaike information criterion (AIC), and by using the rules of thumb recommended by Schermelleh-Engel, Moosbrugger, and Müller [56]. In accordance with those authors, the χ^2^ statistic is highly sensitive to the sample size, and thus not too much emphasis was placed on its significance. To demonstrate good fit, the ratio chi-square by degrees of freedom (χ^2^/df) should not exceed 3.0. Also, CFI values greater than 0.95, GFI values greater than 0.90, and AGFI values greater than 0.85 were considered as having acceptable fit. RMSEA values between 0.05 and0.08 were considered as an adequate fit, with the lower boundary of the confidence interval lower than 0.05 for close fit. For model comparisons, the parsimony indices considered were the RMSEA and the AIC, with lower values of these indices indicating a better model fit. At this stage, different competing models would be compared for the full scale (the SNAddS-6S) and the short scale (the Short SNAddS-6S).

Finally, using the overall sample, we explored the correlation between the SNAddS-6S (and its subscales) and other variables conceptually related to it (with Pearson correlation analyses), as well the differences between men and women on the scores to the scale (with ANOVA analyses), to explore the external validity of the final scale.

## 3. Results

### 3.1. Preliminary Analyses: Common Method Variance

The EFA performed with all the items of understudy constructs showed a 12-factor solution congruent with the number of variables and expected factors included in the study. Moreover, no general factor leads to most of the covariance. The second EFA performed by fixing one factor for extraction explained only 27.56% of the variance. As such, no common method bias was observed.

### 3.2. Stage 1: Reducing the Items and Exploring the Factorial Analysis

#### 3.2.1. Missing Data, Correlation between Items, and Reliability Analyses

The results revealed that no items had more than 10% of missing data (in fact, no missing data were found for the items of the SNAddS-6S, except for item 1, with 0.6% of missing data). No items of the scale displayed correlations lower than 0.20 with half (or more) of the items of the scale. When including all 18 items, high reliability was found (α = 0.91), and no items had low item-to-total correlations. As a result, no items were removed.

#### 3.2.2. Exploratory Factorial Analysis for the Social Networks Addiction (SNAdd) Scale

For the 18 items, the KMO index (0.87) and BTS (χ^2^ = 1612.74; *df* = 153; *p* < 0.001) supported the use of EFA. The EFA showed a five-factor solution with a balanced factorial structure that explained 69% of the variance (Table 1). All the items saturated in the expected factors except for those of salience and tolerance factors, which saturated in a unique factor (the first factor). We have called this higher-order factor “time-management” in accordance with results of the literature in which those two symptoms also merge in a unique factor [57,58,59], and because it includes the two factors related to the difficulty that individuals have in managing time regarding (a) their thoughts about SN, spending most of their time thinking about them, thus reflecting salience, and (b) the time they need for SN to continue to be enjoyable, thus reflecting tolerance. Factor 2 corresponded to mood modification, factor 3 to relapse, factor 4 to withdrawal, and factor 5 to conflict. The global scale (α =0.91) and each of the five factors presented high reliability.

Because the component model of addiction [12] and the subsequent BFAS [27] and SNAddS-6S considered six theoretical factors, to explore the suitability of the six-factor structure, we conducted another EFA by fixing six factors for extraction. Again, the KMO index (0.8) and BTS (χ^2^ = 1612.74; *df* = 153; *p* < 0.001) supported the use of EFA. The six extracted factors explained 73.38% of the variance. All items were loaded congruently to their proposed dimensions (Table 2). Factor 1 represented mood modification, factor 2 relapse, factor 3 withdrawal, factor 4 conflict, factor 5 salience, and factor 6 tolerance. The global scale (α = 0.90) and each of the six factors presented acceptable to high reliability. Consequently, Hypothesis 1 was partially supported.

#### 3.2.3. Exploratory Factorial Analysis for the Short SNAddS-6S

To explore the unidimensionality of the Short SNAddS-6S, we performed an EFA by using the items with higher loadings in each of the five factors found in the EFA without fixing factors for extraction (items 3, 8, 12, 14, and 16), and another EFA by using the items with higher loadings in each of the six factors found in the EFA fixing six factors for extraction (items 2, 3, 8, 12, 14, and 17). In both analyses, the unidimensionality of the short scale was supported.

When using the five items with higher loadings in each of the five factors found in the EFA conducted without fixing factors for extraction, the KMO index (0.74) and BTS (χ^2^ = 113.99; *df* = 10; *p* < 0.001) supported the use of EFA, and the unifactorial structure explained 43.17% of the variance. Reliability was unacceptable (α = 0.66).

When using the six items with higher loadings in each of the six factors found in the EFA conducted by fixing factors for extraction, the KMO index (0.78) and BTS (χ^2^ = 166.46; *df* = 15; *p* < 0.001) also supported the use of EFA, and the unifactorial structure explained 41.73% of the variance. Reliability was good (α = 0.71). In accordance with these results, Hypothesis 2 was partially supported.

### 3.3. Stage 2: Confirming the Factorial Analysis

#### 3.3.1. Confirmatory Factorial Analysis for the SNAddS-6S

**Figure 1:**
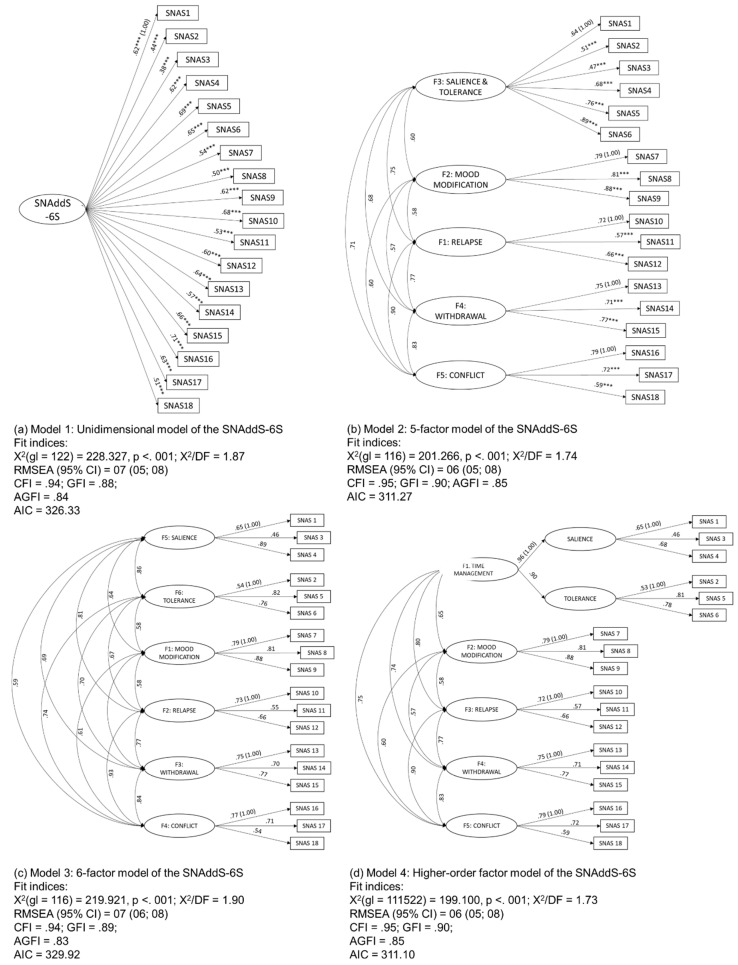
Comparison between the four tested models (**a**–**d**).

To test the unidimensionality or multidimensionality of the large scale (SNAddS-6S), four competing models were compared. In the first model, the unidimensionality of the scale was assumed by performing a single-factor CFA. In the second model, the five-factor structure found in the first EFA conducted was explored. In the third model, we explored the six-factor structure found in the second EFA conducted by fixing six factors for extraction according to the theoretical framework of the Griffiths’ model [12]. In the fourth model, a higher-order factor was explored in which the SNAddS-6S can be partitioned into five factors, with the first being the time-management higher-order factor with two different sub-factors (one corresponding to salience and one to tolerance). This fourth model could conciliate the EFA found with five factors that includes salience and tolerance in one large factor and the theory about six different symptoms of addiction. The four models are presented in Figure 1. The two models with the worse fit were the single-factor model (Model 1; Figure 1a) and the six-factor model (Model 3; Figure 1c), with no acceptable fit indices. The five-factor model (Model 2; Figure 1b) and the higher-order factor model (Model 4; Figure 1d) were the best ones with good fit indices.

#### 3.3.2. Confirmatory Factorial Analysis for the Short SNAddS-6S

**Figure 2:**
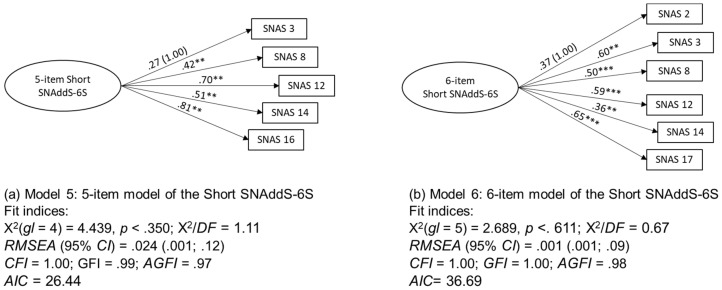
Comparison between the two tested models (**a**,**b**).

To test the unidimensionality of the Short SNAddS-6S, two competing models were compared. In the first one (Model 5), a single-factor CFA was performed with the five items that showed higher loadings when performing the EFA without fixing factors for extraction. In the second one (Model 6), a single-factor CFA was performed with the six items that showed higher loadings when performing the EFA by fixing six factors for extraction, in accordance with the Griffiths component model of addiction [12]. The two models had good to excellent fit indices (Figure 2). The five-item model (Model 5) was the worst short scale model despite its good fit indices. The six-item model (Model 6) was the best, with excellent fit indices.

### 3.4. Exploring the External Validity of the Large and Short SNAddS-6S

To obtain additional evidence of the instrument’s validity, we performed Pearson correlation analyses with the SNAddS-6S, the six-item Short SNAddS-6S (that obtained better fit indices), and the five factors of the higher-order SNAddS-6S (by using the model that obtained better fit indices). As illustrated in Table 3, the expected correlations with other variables of interest were found, thus confirming Hypothesis 4. The SNAdd-6S, the Short SNAddS-6S, and the different factors of the SNAddS-6S positively correlated with SN abuse, loneliness, and social anxiety, and negatively so with life satisfaction and self-esteem.

**Figure 3:**
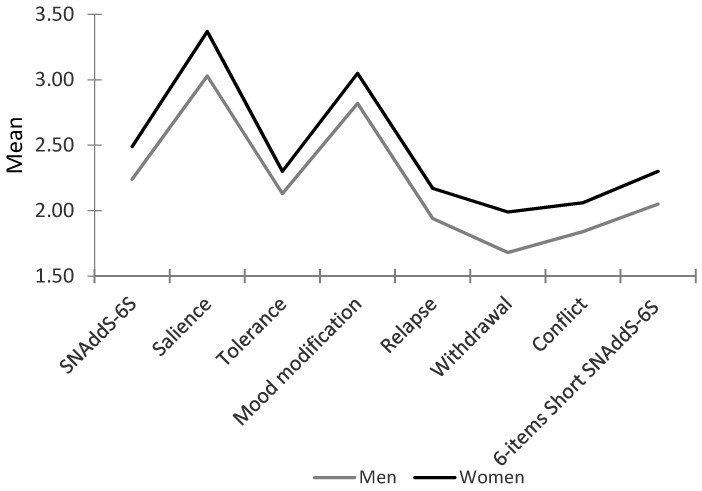
Differences between men and women in the SNAddS-6S and its factors and in the Short SNAddS-6S.

One-way and multifactorial ANOVA analyses performed confirmed Hypothesis 5. The one-way ANOVA analyses performed demonstrated higher scores for women in both the six-item Short SNAddS-6S (*F*(1,361) = 8.56, *p* < 0.01) and the SNAddS-6S (*F*(1,361) = 8.58, *p* < 0.01). Moreover, the multifactorial ANOVA analysis performed by introducing the six factors of the SNAdd-6S as dependent variables, and gender as factor, has shown higher significant differences (*F* (6,355) = 2.30, *p* < 0.05) with higher scores for women in all the factors of the scale (*F*_salience_ (1,361) = 10.94, *p* <0.001; *F*_relapse_ (1,361) = 3.83, *p* < 0.05; *F*_withdrawal_ (1,361) = 7.93, *p* < 0.01; and *F*_conflict_ (1,361) = 4.04, *p* < 0.05), except for mood modification (*F* (1,361) = 2.55, *ns*) and tolerance (*F* (1,361) = 2.64, *ns*), for which no differences were found (Figure 3).

## 4. Discussion

There has been an increase in the use of the Internet and SN [15] and individuals cannot now imagine their lives without SN. But this increase of SN use also has some potential risks, such as addiction, especially for young people [29]. Nonetheless, to the best of our knowledge, there is not any multifactorial SNA scale properly validated in the scientific literature based on the six core components of addiction described by Griffiths [12]. Consequently, there is a need to develop a comprehensive and psychometrically sound scale in which those symptoms are reflected to detect possible addiction or problematic SN use in individuals and to carry out research on the potential predictors of SNA and its different factors.

### 4.1. The SNAddS-6S, a Multidimensional Valid and Reliable Scale, and the Short SNAddS-6S, a Unidimensional Valid and Reliable Scale

The results of our analyses confirmed the multidimensionality of SNA. The results of the EFA and CFA performed confirmed a robust adjustment for the higher-order five-factor structure. The six-factor and one-factor solutions presented unacceptable fit indices, while the five-factor solution presented correct but poorer fit indices in comparison to the higher-order factor solution. Thus, Hypothesis 1 was partially supported. The 18 items of the scale had saturated in five factors that approximately correspond to those highlighted in the component model of addiction [12]. The results of the EFA and CFA were congruent with this model, and the five dimensions identified corresponded, in this order of relevance, to the time-management symptom of addiction (which includes two different related symptoms that emerged as sub-factors of the time-management higher-order factor: Salience and tolerance), the mood modification symptom, the relapse symptom, the withdrawal symptom, and finally the conflict symptom. Thus, this large scale and its different factors are especially relevant when researchers or practitioners want to know more about the determinants of addiction, considering that salience and tolerance saturated into a unique factor. This merging of the components of salience and tolerance into a higher factor called time-management is congruent with previous literature [57,58]. The time-management higher factor represents the difficulty that individuals have in managing time regarding (a) their thoughts about SN, spending most of their time thinking about them, and (b) the time they need for SN to continue to be enjoyable. In this sense, Chang and Law [57], in the addiction to Internet context, also found that both factors—salience and tolerance—merged into a single unique factor they called “time management”; and Charlton and Danforth [58] classified criteria of Internet game addiction into core criteria and peripheral criteria, the latter including both salience and tolerance together. In the same way, salience and tolerance presented the higher correlation in a study investigating the context of game addiction [59]. In future studies regarding behavioral and Internet related addiction, the relation between salience and tolerance must be explored.

Moreover, the Short SNAddS-6S was demonstrated to be a unidimensional reliable and valid scale. Both EFA and CFA analyses confirmed a robust adjustment for the unifactorial structure when using six items with higher loadings in each factor found in the EFA performed by fixing six factors for extraction. The six-item short scale has shown better fit indices than the five-item short scale. Moreover, the five-item short scale demonstrated low reliability, with a low Cronbach alpha value. Thus, the six-items short scale—but not the five-item short scale—can be used by researchers and practitioners. Another reason to adopt the six-item short scale is because it corresponds to the inclusion of one item of each of the six core components of addiction, which then represents all of them in this short scale form. In any case, the use of the Short SNAddS-6S could be relevant in studies and interventions in which a valid, rapid, and cheap evaluation of SNA is needed, without the need for the evaluation of each of the symptoms of addiction separately.

The external validity of the SNAddS-6S and the Short SNAddS-6S were tested across the relation of the scales with different measures. The pattern of relations among the SNAddS-6S (and the Short SNAddS-6S) and its factors with other psychological variables provided strong evidence for construct validity. As expected, and in congruence with previous literature, we found the SNAddS-6S and each of its factors to be correlated with social network abuse. In accordance with different authors that argued and demonstrated the intrinsic relation between abuse and addiction [28,29,30], the results show that the more individuals presented addictive symptoms to SN (high scores on the SNAddS-6S and the Short SNAddS-6S) or the more their scores on the different dimensions of SN addiction were high, the more they abuse SN. Moreover, women reported higher scores on the different dimensions of the scale, as per the assumption that they used the Internet more for social interaction and that they are more prone to be implicated in addiction involving social interaction than men [27,46]. However, results about sex differences in SNA are sometimes incongruent, with some studies indicating higher levels of addiction in men, and others in women [60,61]. Future researchers should therefore focus on the plausible interaction between age and sex or between other socio-demographic and personal variables to explain the sex differences.

The expected relations also emerged between the SNAddS-6S and loneliness. There are several authors who have claimed that Internet addiction and SNA are related to loneliness [36,37,38]. Previous studies discovered that loneliness was a relevant variable associated with Internet addiction and its different symptoms [37] and highlighted the potential risk for lonely individuals to develop Internet (or SN) addiction because they might prefer online social interaction [62]. In accordance with the results of previous literature, the SNAddS-6S and the Short SNAddS-6S positively correlated with loneliness. Thus, the results showed that the more individuals presented addictive symptoms to SN (high scores on the SNAddS-6S and the Short SNAddS-6S) or the more their scores on the different dimensions of SNA were high, the more they felt lonely.

The relation between the SNAddS-6S and social anxiety was also confirmed. Previous literature has demonstrated that individuals with high levels of social anxiety tended to be addicted to the Internet and SN, perhaps because of the reduction of fear to the interaction that the communication mediated by a computer provides [31,32,33,35,63]. In accordance with this literature, our SNAddS-6S, Short SNAddS-6S, and the different factors of the SNAddS-6S correlated positively with social anxiety.

Moreover, the relation between the SNAddS-6S and the Short SNAddS-6S with life satisfaction was also congruent with the reviewed literature showing that Internet addiction and SNA are related to poorer levels of satisfaction with life [37,39,40,42,43,44]. Accordingly, our results showed that the scores on the SNAddS-6S (or on the different factors) were higher, the more they were dissatisfied with life.

Finally, the relation with self-esteem was also coherent with previous research [9,37,43,45], and the more individuals were addicted to the Internet and SN, the lower their self-esteem levels.

### 4.2. Limitations and Future Research

Although we had an adequate sample size for the validation of a sound scale, it should be noted that the data are cross-sectional, and that the findings cannot be generalized due to the non-representative sample. Consequently, future research should conduct cross-cultural studies.

Moreover, the sample was predominantly composed of women. While there is no reason to think that the results would be different in a more gender-balanced sample, future research should explore the scale in a more generalizable manner. 

Additionally, although the SNAddS-6S can be used by practitioners in their routine clinical practice as well as by researchers, in the present study the authors did not examine the cutoff scores for SNA. As such, future studies should explore the adequate cutoff values for the categorization of SNA. In this sense, “a liberal approach would entail the use of a polythetic scoring scheme” [27] (applied to our scale, scoring 3 or above on at least four of the six factors of the SNAddS-6S, or scoring 3 or above on at least four of the six items of the Short SNAddS-6S). This kind of approach is consistent with the fact that people are usually categorized as addicted when they fulfil a given number of criteria [13].

Finally, it could be argued that the scale lacked three criterions included in the DSM-V for Internet gaming disorder (IGD). In this sense, van den Eijdnen and colleagues [64] developed a Social Media Disorder Scale which included an item for each of the nine diagnostic criterions of the DSM-V for IGD. The authors argued that IGD and social media disorder are two forms of Internet addiction, and social media addiction and IGD scales should include the same set of diagnostic criteria. In the DSM-V, in addition to the six-core components of addiction included here, three other diagnostic criteria are found, namely “problems,” “deception,” and “displacement.” Although our scale focuses only on the six core components of addiction, it could be interesting in the future to expand this scale by adding three items for each of these three diagnostic criteria included in the DSM-V for researchers that will be interested in investigating these criteria further. The principal differences between our expanded scale and the Social Media Disorder Scale [64] will be that (a) our scale will focus on SN and not on social media; (b) in our scale participants respond with a Likert scale and not with yes/no answers; and (c) our scale was validated for the multifactorial (large scale) and unifactorial structure (short scale).

## 5. Conclusions

Our analyses provide evidence for the validity and reliability of both the large multidimensional SNAddS-6S, with its different factors, and the Short SNAddS-6S. Depending on whether researchers and professionals need to obtain information about the different factors of SNA, and whether they need a rapid and short version, they can choose the form of the scale best suited to their situation. In a society in which the use and abuse of the Internet and SN have become increasingly high, and taking into account that to our knowledge there is not any validated scale for measuring SNA on the basis of the six core components of addiction [12], this scale could be of relevance for researchers and practitioners to assess the extent to which individuals suffer from SNA and to study the potential predictors and risks of such addiction.

## Figures and Tables

**Table 1:** Results of the exploratory factor analysis (EFA) of the SNAddS-6S: Factors loading and reliability estimates.

Items	Highest Loading for Each One of the Five Factors				
	F1	F2	F3	F4	F5
SNAdd 1	0.642				
SNAdd 2	0.633				
SNAdd 3	0.662				
SNAdd 4	0.531				
SNAdd 5	0.638				
SNAdd 6	0.548				
SNAdd 7		0.863			
SNAdd 8		0.882			
SNAdd 9		0.864			
SNAdd 10			0.409		
SNAdd 11			0.815		
SNAdd 12			0.865		
SNAdd 13				0.760	
SNAdd 14				0.803	
SNAdd 15				0.662	
SNAdd 16					0.808
SNAdd 17					0.754
SNAdd 18					0.669
Scale reliability estimates					
Cronbach’s Alpha values	0.81	0.89	0.80	0.80	0.78
Percentage of explained variance	14.77	14.42	13.61	13.46	12.74

**Table 2:** Results of the EFA of the SNAddS-6S when fixing six factors for extraction: Factors loading and reliability estimates.

Items	Highest Loading for Each One of the Six Factors					
	F1	F2	F3	F4	F5	F6
SNAdd 1						0.659
SNAdd 2					0.694	
SNAdd 3						0.732
SNAdd 4						0.667
SNAdd 5					0.634	
SNAdd 6					0.629	
SNAdd 7	0.865					
SNAdd 8	0.883					
SNAdd 9	0.862					
SNAdd 10		0.400				
SNAdd 11		0.806				
SNAdd 12		0.859				
SNAdd 13			0.779			
SNAdd 14			0.829			
SNAdd 15			0.472			
SNAdd 16				0.797		
SNAdd 17				0.801		
SNAdd 18				0.668		
Scale reliability estimates						
Cronbach’s Alpha values	0.89	0.80	0.80	0.78	0.75	0.70
Percentage of explained variance	14.36	12.87	12.25	12.11	11.25	10.52

**Table 3:** Correlation analyses.

	SN Abuse	Loneliness	Social Anxiety	Life Satisfaction	Self-Esteem
SNAddS-6S	0.54 ***	0.28 ***	0.31 ***	−0.27 ***	−0.35 ***
Time-management higher-order factor	0.49 ***	0.16 ***	0.20 ***	−0.13 ***	−0.21 ***
Salience sub-factor	0.48 ***	0.10 ***	0.16 ***	−0.11 ***	−0.18 ***
Tolerance sub-factor	0.40 ***	0.18 ***	0.20 ***	−0.12 ***	−0.19 ***
Mood modification factor	0.42 ***	0.32 ***	0.34 ***	−0.34 ***	−0.40 ***
Relapse factor	0.33 ***	0.24 ***	0.22 ***	−0.17 ***	−0.24 ***
Withdrawal factor	0.41 ***	0.16 ***	0.23 ***	−0.19 ***	−0.26 ***
Conflict factor	0.41 ***	0.22 ***	0.22 ***	−0.25 ***	−0.28 ***
Six-items Short SNAddS-6S	0.50 ***	0.28 ***	0.30 ***	−0.27 ***	−0.36 ***

*** *p* < 0.001.

## Supplementary Materials

The following are available online at https://www.mdpi.com/2254-9625/10/3/56/s1, Table S1: Spanish version of the SNAddS-6S.
