# Supplementary material for: Development and Validation of the Social Network Addiction Scale (SNAddS-6S)
Source: Eur J Investig Health Psychol Educ. 2020 Jul 26;10(3):763–78. doi: 10.3390/ejihpe10030056 (PMC8314296; doi:10.3390/ejihpe10030056)
Supplement: Supplementary file 1 [file ejihpe-10-00056-s001.pdf]

Table S1. Spanish version of the SNAddS-6S

Items	
SNAdd 1	¿Has pasado mucho tiempo pensando en conectarte a las Redes Sociales o planeando su uso?
SNAdd 2	¿Has pasado más tiempo utilizando las Redes Sociales de lo que inicialmente pretendías?
SNAdd 3	¿Has pensado sobre cómo podrías pasar más tiempo conectado a las Redes Sociales?
SNAdd 4	¿Has pasado mucho tiempo pensando en lo que ha sido publicado en las Redes Sociales?
SNAdd 5	¿Has sentido la necesidad de usar las Redes Sociales cada vez más?
SNAdd 6	¿Has sentido que cada vez necesitabas más tiempo para usar las Redes Sociales y que te siguieran resultando igualmente placenteras?
SNAdd 7	¿Has usado las Redes Sociales para olvidarte de problemas personales?
SNAdd 8	¿Has usado las Redes Sociales para reducir sentimientos de culpa, ansiedad, impotencia y/o depresión?
SNAdd 9	¿Has usado las Redes Sociales para reducir tu inquietud o preocupación?
SNAdd 10	¿Otras personas te han aconsejado reducir el uso de las Redes Sociales, pero no les has hecho caso?
SNAdd 11	¿Has intentado reducir el uso de las Redes Sociales sin éxito?
SNAdd 12	¿Has decidido usar las Redes Sociales con menos frecuencia, pero no lo has conseguido?
SNAdd 13	¿Has llegado a estar inquieto o preocupado si te han prohibido usar las Redes Sociales?
SNAdd 14	¿Has llegado a estar irritable si te han prohibido usar las Redes Sociales?
SNAdd 15	¿Te has sentido mal si, por diferentes razones, no has podido iniciar sesión en las Redes Sociales durante algún tiempo?
SNAdd 16	¿Has usado las Redes Sociales tanto que ha tenido un impacto negativo en tu trabajo/estudios?
SNAdd 17	¿Has dado menos prioridad a tus aficiones, actividades de ocio y/o ejercicio por culpa de las Redes Sociales?
SNAdd 18	¿Has ignorado a tu pareja, familia o amigos por culpa de las Redes Sociales?
